# Supplementary material for: Novel Highly Conductive and Transparent Graphene-Based Conductors
Source: Adv Mater. 2012 Apr 25;24(21):2844–9. doi: 10.1002/adma.201200489 (PMC3715101; doi:10.1002/adma.201200489)
Supplement: Supplementary file 1 [file adma0024-2844-SD1.pdf]

Copyright WILEY-VCH Verlag GmbH & Co. KGaA, 69469 Weinheim, Germany, 2012.

ADVANCED MATERIALS

Supporting Information

for *Adv. Mater.*, DOI: 10.1002/adma.201200489

Novel Highly Conductive and Transparent Graphene-Based Conductors

*Ivan Khrapach, Freddie Withers, Thomas H. Bointon, Dmitry K. Polyushkin, William L. Barnes, Saverio Russo, and Monica F. Craciun **

Supporting Information

Thickness determination of pristine few-layer graphene on glass

Our experiments are based on few layer graphene (FLG) deposited on both SiO_2/Si and glass substrates (100 μm thick). The thickness of FLG on SiO_2/Si was determined by optical contrast using 280nm SiO_2 and green light, following a well-established technique described in detail in Ref. [1]. Contrary to FLG on SiO_2/Si, where the optical contrast can be maximized by using the appropriate thickness of SiO_2 and the wavelength of light, for FLGs deposited on glass the contrast varies only slightly with the wavelength of light. Therefore, the thickness of FLG on glass was determined by analyzing the intensity of the FLG optical micrographs taken with white light (see Figure 1a). Figure 1b shows the optical contrast (C) of FLG on glass, where C = $(I_f - I_s)/I_s \times 100\%$ is the relative shift of the intensity of the flake (I_f) with respect to the intensity of the substrate (I_s). It is apparent that step-like changes are visible with increasing optical contrast. As we will show, Raman spectroscopy demonstrates that each step corresponds to an increase by 1 in the number of layers.

Raman spectroscopy offers a reliable approach to count the number of layers (N) in FLG on SiO_2/Si substrates, based on the ratios of the intensities of the G peak and the Si peak (I_G/I_{Si}). It was recently shown that I_G/I_{Si} increases monotonically and discretely with N due to an increase of the intensity of the G peak and to a decrease of the intensity of the Si peak [2, 3]. In order to use this method, we placed the glass substrate on top of a SiO_2/Si substrates with the FLGs facing the surface of SiO_2 (see Figure 1c). Figure 1d shows I_G/I_{Si} for FLG with different N determined from the optical contrast. I_G/I_{Si} increases monotonically with increasing N as expected. This analysis shows that optical contrast and Raman spectroscopy provide two independent methods which can be successfully used as a tool for determining the thickness of FLG on glass.

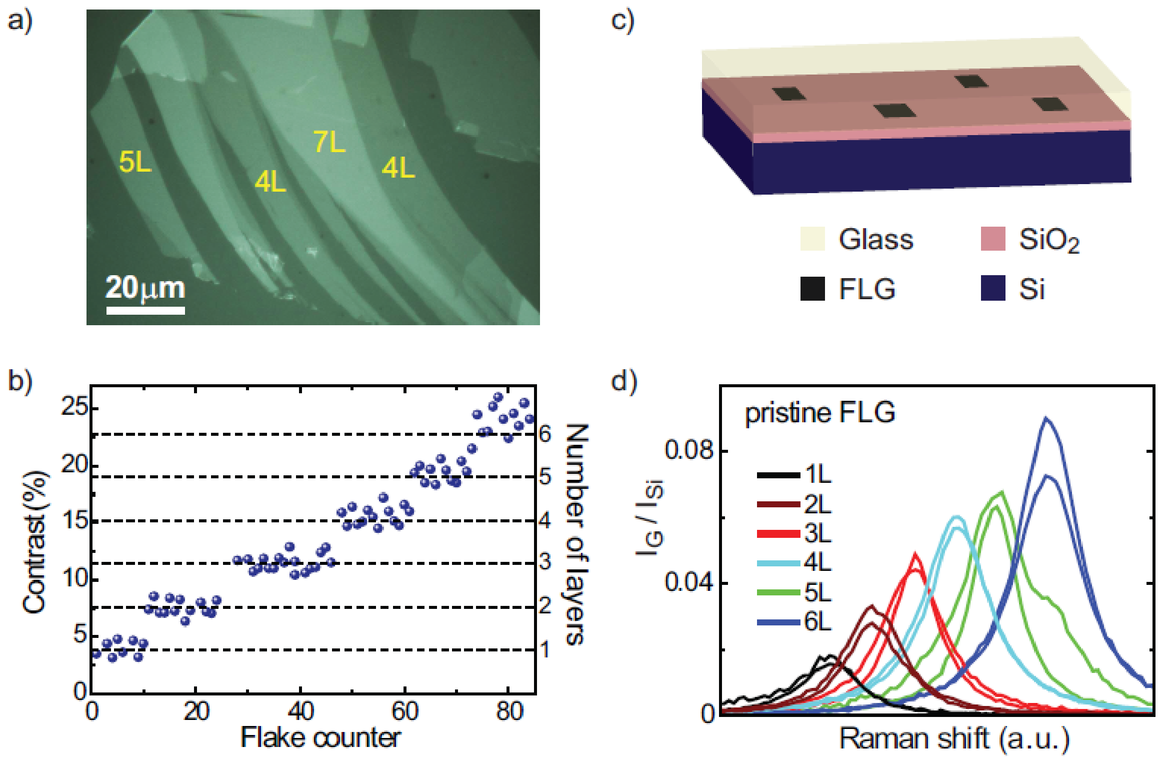

FIG. 1: a) Optical micrograph of a FLG flake on glass taken with white light. The glass substrate was suspended in air. b) The optical contrast of FLG on glass plotted for 85 flakes. c) Schematic of the glass substrate with FLG on top of the SiO_2/Si. d) The ratios of the intensities of the G peak and the Si peak (I_G/I_{Si}). The curves are shifted on the x axis for clarity.

Homogeneity and stability in air of FeCl3-intercalated five-layer devices

We use Raman spectroscopy (see Experimental section) to characterize the homogeneity of the $FeCl_3$ intercalation in FLGs after the fabrication of electrical contacts. Figure 2 shows a micrograph picture of the 5L intercalated device discussed in Fig. 3 in the main text. A direct comparison of the Raman spectra measured at 10 different locations on the device, as highlighted in the picture, shows no appreciable variation in either intensity or position of the Raman peaks. This demonstrates that our intercalated flakes have a high homogeneity, i.e. the structure is the same at any point of the flake. In particular, the peak at 1590 cm^{-1} is the G-

peak and its presence implies that at least one graphene plane has no adjacent layers of $FeCl_3$. This graphene sheet is likely to be the top layer, since rinsing of samples in acetone is known to remove $FeCl_3$ from the surface of the flakes [4]. The peaks at 1612cm^{-1} and at 1625cm^{-1} correspond to a uniform layer of intercalant on one side of the graphene and on both sides of the graphene, respectively. The fact that the flake has three single-layer intercalated planes and one bilayer plane is also supported by Shubnikov - de Haas oscillations of the longitudinal conductivity in strong magnetic field (see Fig 1 in the main text). The crystal structure of these flakes is reported in Fig. 2a and it is homogeneous throughout the flake.

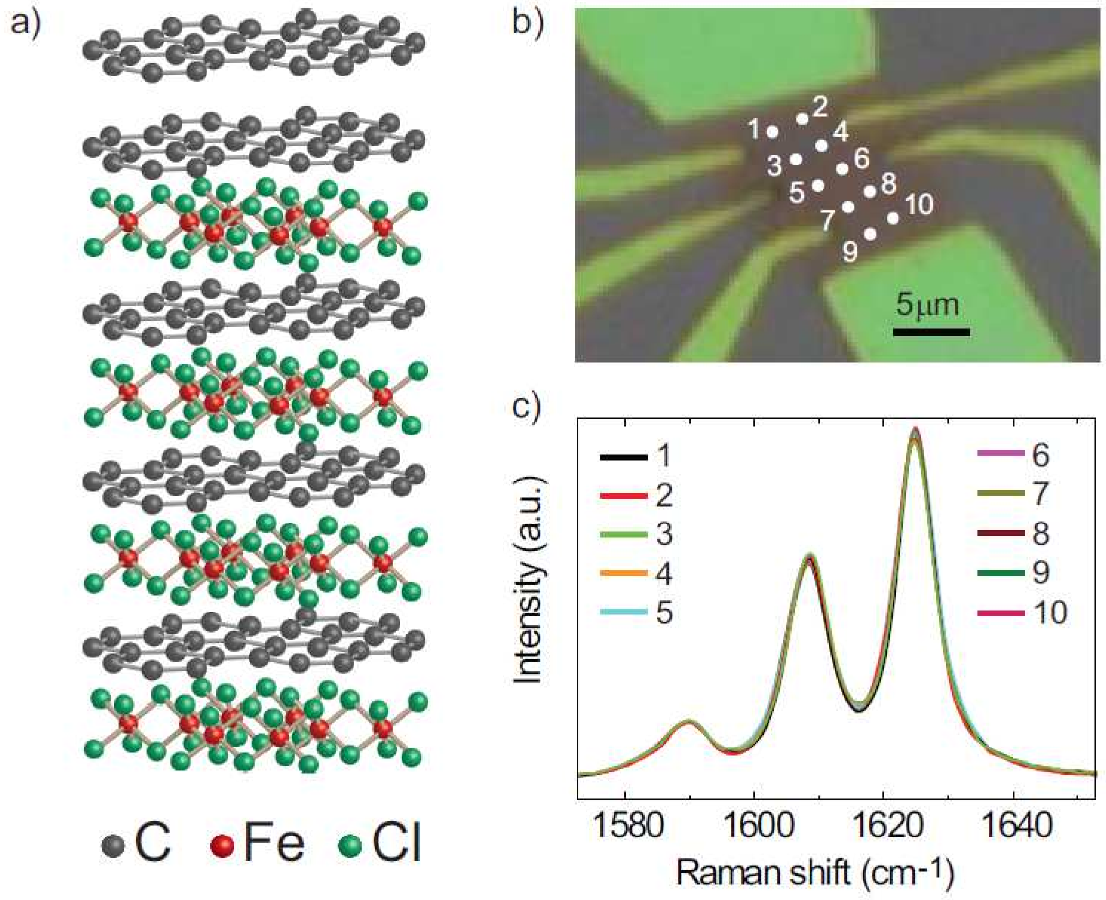

FIG. 2: a) Schematic crystal structure of a 5L $FeCl_3$-FLG after device fabrication. b) Optical image of 5L $FeCl_3$-FLG. c) Raman spectra of 5L $FeCl_3$-FLG devices taken at different locations as indicated in b).

Another function required by a transparent conductor is stability in air. Therefore, we have studied the air stability of these interlacated FLGs as a function of time. Fig. 3 shows a comparison between Raman spectra collected at different positions on a $FeCl_3$-5L sample, after keeping the samples for 3 months (Fig. 3a) and one year (Fig. 3b) in air. It is apparent that the spectra show no appreciable change. This demonstrates the stability in air of $FeCl_3$-5L intercalated devices.

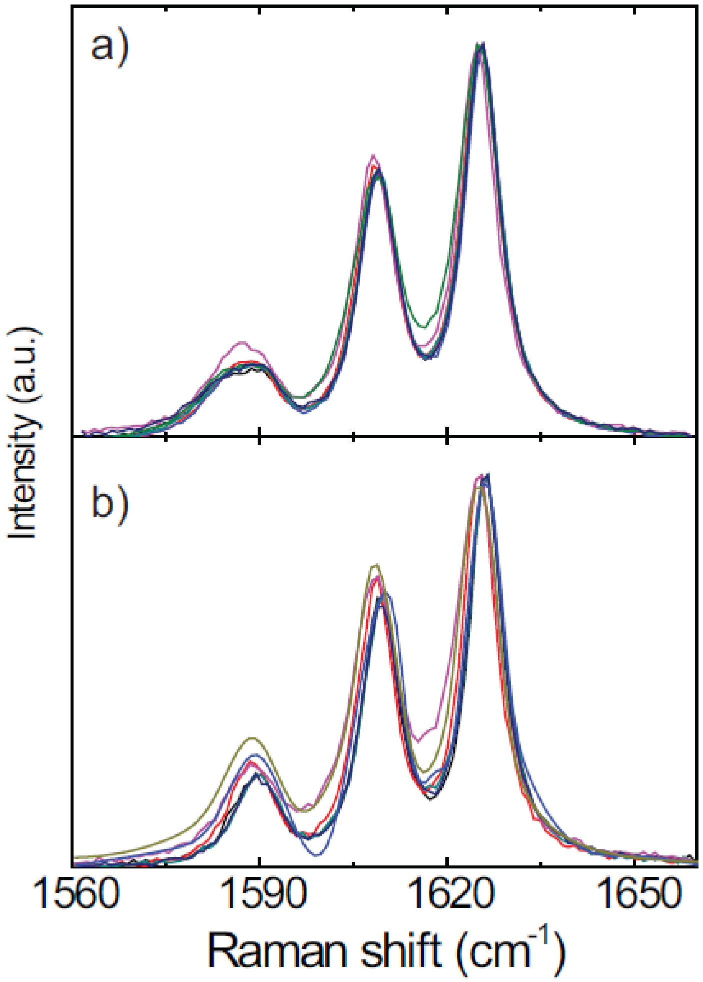

FIG. 3: Raman spectra of a typical 5L $FeCl_3$-FLG devices taken at different locations after 3 months (a) and after one year (b).

Comparison between FeCl3-intercalated four- and five-layer devices

Figure 4 (a) and (b) show a comparison between the electrical properties of several 4L and 5L intercalated FLGs devices. At room temperature the 5L samples typically exhibit lower values of sheet resistance than the 4L samples (see Fig 4a). Furthermore we have measured the temperature dependence for 3 intercalated 5L samples and they all show lower sheet resistance than the 4L samples over all the investigated temperature range (see Fig 4b). Figure 4c shows that the Raman spectra of 4L intercalated FLGs devices is similar to the one of 5L intercalated devices, i.e. they both show the presence of pristine G, G_1 and G_2 peaks. This suggests that 4L and 5L intercalated devices have similar crystal structures.

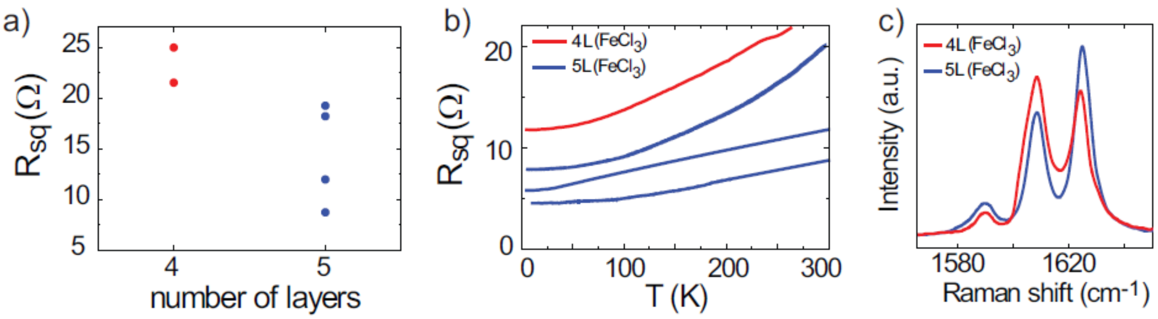

FIG. 4: a) Room temperature square resistance for 4L and 5L $FeCl_3$-FLG. b) Temperature dependence of the square resistance for 4L and 5L $FeCl_3$-FLG. c) Raman spectra of 4L and 5L $FeCl_3$-FLG devices.

Hall versus total carrier concentration in a layered structure

The intercalation of $FeCl_3$ between the graphene planes originates parallel conductive layers, each with a specific charge carrier concentration (n_i) and mobility (μ_i). At low-magnetic fields, the classical Hall effect measured for N parallel conductive layers is a function of the Hall charge carrier concentration $n_H = \sum_i n_i^2 \mu_i^2 / \sum_i n_i \mu_i^2$ [5]. We note that n_H is smaller than the total carrier concentration $n_{tot} = \sum_i n_i$. This is apparent when considering

$\frac{n_H}{n_{tot}} = \frac{n_{eff}}{\sum_i n_i} = \frac{n_i^2 \mu_i^2}{(\sum_i n_i)(\sum_i n_i^2 \mu_i^2)} = \frac{n_i^2 \mu_i^2}{\sum_i n_i^2 \mu_i^2 + \sum_{i \neq i} n_j n_i \mu_i^2}$ which is less than unity. The relation n_H/n_{tot}<1 together with Shubnikov-de Haas oscillations and Raman spectroscopy is used to identify the number of parallel conductive layers in $FeCl_3$-FLG.

Cyclotron mass of charge carriers in bilayer graphene

The semiclassical expression for the cyclotron mass of charge carriers is $m_c = (\hbar^2/2\pi)\partial S(E)/\partial E$ with $S(E)=\pi k^2$ the area enclosed by the orbits of the charge carriers in *k*-space [6]. The energy dispersion of bilayer graphene is: $E(k) = \pm\frac{\gamma_1}{2} \pm \sqrt{\frac{\gamma_1^2}{4} + (\hbar v_F k)^2}$ considering non-zero only the first neighbour hopping parameters γ_0 (in-plane) and γ_1 (interlayer) [7]. The cyclotron mass is given by: $m_c = \frac{\hbar^2}{2\pi} 2\pi k \frac{\partial k}{\partial E} = \frac{\sqrt{\frac{\gamma_1^2}{4} + (\hbar v_F k)^2}}{v_F^2}$. For bilayer graphene electron(hole) charges, m_c can be expressed as a function of the carrier concentration (n) as follows: $m_c = \sqrt{(\hbar v_F)^2 \pi n + (\gamma_1/2)^2}/v_F^2$ with $n = \sqrt{k_F^2/\pi}$ [7].

[1] M. F. Craciun, S. Russo, M. Yamamoto, J. B. Oostinga, A. F. Morpurgo, S. Tarucha, *Nature Nanotech.* **2009**, *4*, 383.

[2] Y. K. Koh, M.-H. Bae, D. G. Cahill, E. Pop, *ACS Nano* **2011**, *5*, 269.

[3] S.H. Jhang, M. F. Craciun, S. Schmidmeier, S. Tokumitsu, S. Russo, M. Yamamoto, Y. Skourski, J. Wosnitza, S. Tarucha, J. Eroms, C. Strunk, *Phys. Rev. B* **2011**, *84*, 161408(R).

[4] N. Kim, K. S. Kim, N. Jung, L. Brus, P. Kim, *Nano Lett.* **2011**, *11*, 860.

[5] J. H. Davies, The physics of low-dimentional semiconductors (Cambridge Univ. Press, Cambridge, 1998)

[6] N. W. Ashcroft, N. D. Mermin, Solid State Physics (Saunders College, Philadelphia, 1976)

[7] E. McCann, V. I. Fal'ko, *Phys. Rev. Lett.* **2006**, *96*, 086805.
